# Photosensitizer Nanoparticles Boost Photodynamic Therapy for Pancreatic Cancer Treatment

**DOI:** 10.1007/s40820-020-00561-8

**Published:** 2021-01-04

**Authors:** Huanyu Yang, Renfa Liu, Yunxue Xu, Linxue Qian, Zhifei Dai

**Affiliations:** 1 Department of Ultrasound, Beijing Friendship Hospital, Capital Medical University, No. 95 Yongan Road, Xicheng District, Beijing, 100050 People’s Republic of China; 2 Department of Biomedical Engineering, College of Engineering, Peking University, No. 5 Yiheyuan Road, Haidian District, Beijing, 100871 People’s Republic of China

**Keywords:** Photodynamic therapy, Photosensitizer, Nanoparticle, Pancreatic cancer, Combined therapy

## Abstract

Patients with pancreatic cancer (PCa) have a poor prognosis apart from the few suitable for surgery. Photodynamic therapy (PDT) is a minimally invasive treatment modality whose efficacy and safety in treating unresectable localized PCa have been corroborated in clinic. Yet, it suffers from certain limitations during clinical exploitation, including insufficient photosensitizers (PSs) delivery, tumor-oxygenation dependency, and treatment escape of aggressive tumors. To overcome these obstacles, an increasing number of researchers are currently on a quest to develop photosensitizer nanoparticles (NPs) by the use of a variety of nanocarrier systems to improve cellular uptake and biodistribution of photosensitizers. Encapsulation of PSs with NPs endows them significantly higher accumulation within PCa tumors due to the increased solubility and stability in blood circulation. A number of approaches have been explored to produce NPs co-delivering multi-agents affording PDT-based synergistic therapies for improved response rates and durability of response after treatment. This review provides an overview of available data regarding the design, methodology, and oncological outcome of the innovative NPs-based PDT of PCa.

## Highlights

- Current clinical studies of photodynamic therapy against pancreatic cancer are reviewed.
- Advantages of nanoparticles in boosting therapeutic efficacy of photodynamic therapy for pancreatic cancer treatment are summarized.
- Challenges and outlook for the future development of nanoparticles-based photodynamic therapy in human are discussed.

## Graphical abstract

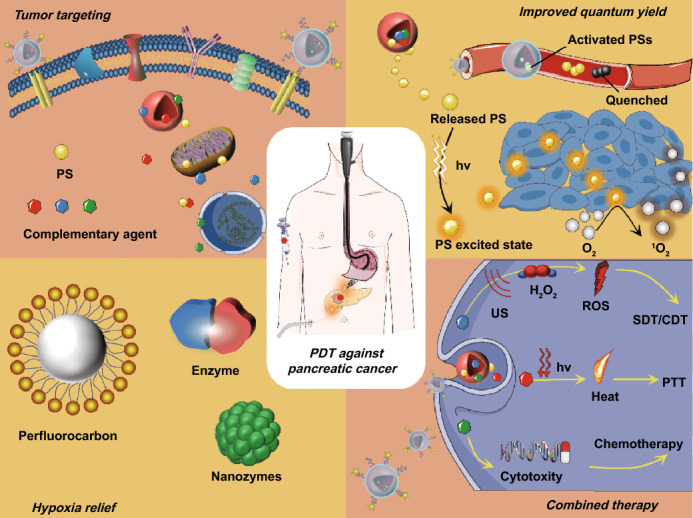

## Introduction

Pancreatic cancer (PCa) remains a lethal disease for which 5-year survival rate of all diagnostic stages combined is only about 9% [1]. For localized resectable tumors, surgery followed by adjuvant chemotherapy (gemcitabine plus capecitabine) is the standard of care, and the 5-year survival rate in these patients is about 30% [2]. However, more than 80% of patients have locally advanced or metastatic disease at diagnosis and are unsuitable for curative surgical resection [3]. Chemotherapy is the main therapeutic option for inoperable patients. However, PCa is relatively chemotherapy-refractory, even with the most aggressive FOLFIRTNOX regimen (folinic acid, 5-fluorouracil, irinotecan, and oxaliplatin), the median overall survival of patients with metastatic disease does not exceed one year (11.1 months), at the cost of cumulative systemic toxicity [4].

Photodynamic therapy (PDT) has emerged as a potential alternative therapy for unresectable patients due to its effectiveness against chemo- and radio-resistant cells [5, 6]. PDT relies on the accumulation of photosensitizer (PS) in tumors, which upon light irradiation transfers absorbed photon energy or excited electrons to surrounding oxygen to generate singlet oxygen (^1^O_2_) and other forms of reactive oxygen species (ROS) [7, 8]. The generated ROS can directly trigger intrinsic apoptotic pathways associated with oxidative damage to mitochondria [9–11]. Thus, PDT can directly activate the late stages of the apoptotic program that bypasses many cell death signaling pathways.

PDT has been used as part of the palliative care for PCa and exhibited potential superiority in existing clinical trials. Due to the special position and vital function of pancreas, the pancreatic cancer surgery is the most difficult to conduct and operative mortality varied between 0 and 10% [12]. On the contrary, PDT can be conducted in a minimally invasive way by inserting optical fiber into the tumor to deliver light. Since the PSs can be selectively targeted to tumors, the potential damage to vital tissues can be minimized. However, it suffers from certain limitations during clinical exploitation, including insufficient PSs delivery, tumor-oxygenation dependency, and treatment escape of aggressive tumors [13]. Recently, PDT-related treatments mediated by nanoparticles (NPs) have been proven by basic researches to be able to overcome these obstacles [14]. NPs-based strategy is attractive for PDT of PCa. On the one hand, the encapsulation of PSs in NPs could improve the efficacy of PDT by improving the solubility and stability of PSs, increasing targeted delivery of PSs in tumors, and relieving the tumor hypoxia by oxygen supplying. On the other hand, NPs can also be used to load other therapeutic agents for PDT-based synergistic therapy [5]. The present review analyzed the principal application strategies, mechanism of action, and available experimental data of NPs-mediated PDT against PCa.

## Clinical Status of PDT for Pancreatic Cancer

PDT has been reported to be effective and safe for the management of nonmetastatic locally advanced pancreatic cancer in several pilot phase I/II studies, where laser light was delivered along the optical fibers positioned percutaneously through the anterior abdominal wall or via transgastric/transduodenal puncture under endoscopic ultrasound (EUS) guidance, to overcome the limitations of light attenuation in penetrating biological tissues. The first clinical study of PDT for PCa was conducted by Bown et al. in 16 patients, using mTHPC (meta-Tetra(hydroxyphenyl)chlorin), and the fiber was inserted percutaneously using a combination of ultrasound imaging and computed tomography (CT) guidance [12]. Substantial tumor necrosis was detected with CT following PDT treatment and all the patients except two left hospital in 10 days. In a further phase I/II clinical study, mTHPC was replaced with verteporfin (liposomal benzoporphyrin derivative) for its reduced skin photosensitivity and longer wavelength absorption peak with deeper tissues penetration [15]. Due to the proximity of the endoscope to the pancreas, optical fiber can be inserted into the tumor via a transgastric or transduodenal approach under EUS guidance. Choi et al. described the first application of EUS-guided PDT (EUS-PDT) using chlorin e6 (Ce6) derivative in four patients with locally advanced pancreaticobiliary cancer and demonstrated the technique feasibility of EUS-PDT [16]. In another phase I study of EUS-PDT using porfimer sodium, 6 of 12 (50%) patients showed increased pancreatic tumor necrosis after EUS-PDT [17]. These studies confirm that PDT can achieve controllable tumor necrosis with low adverse event profile, although precautions should be taken for tumors invading the duodenal wall or gastroduodenal artery. It is of value for locally advanced disease in local tumor control and combined application before or after chemoradiotherapy. Besides, some locally advanced patients can become qualified for surgery after PDT due to tumor downstaging [15, 17].

**Fig. 1:**
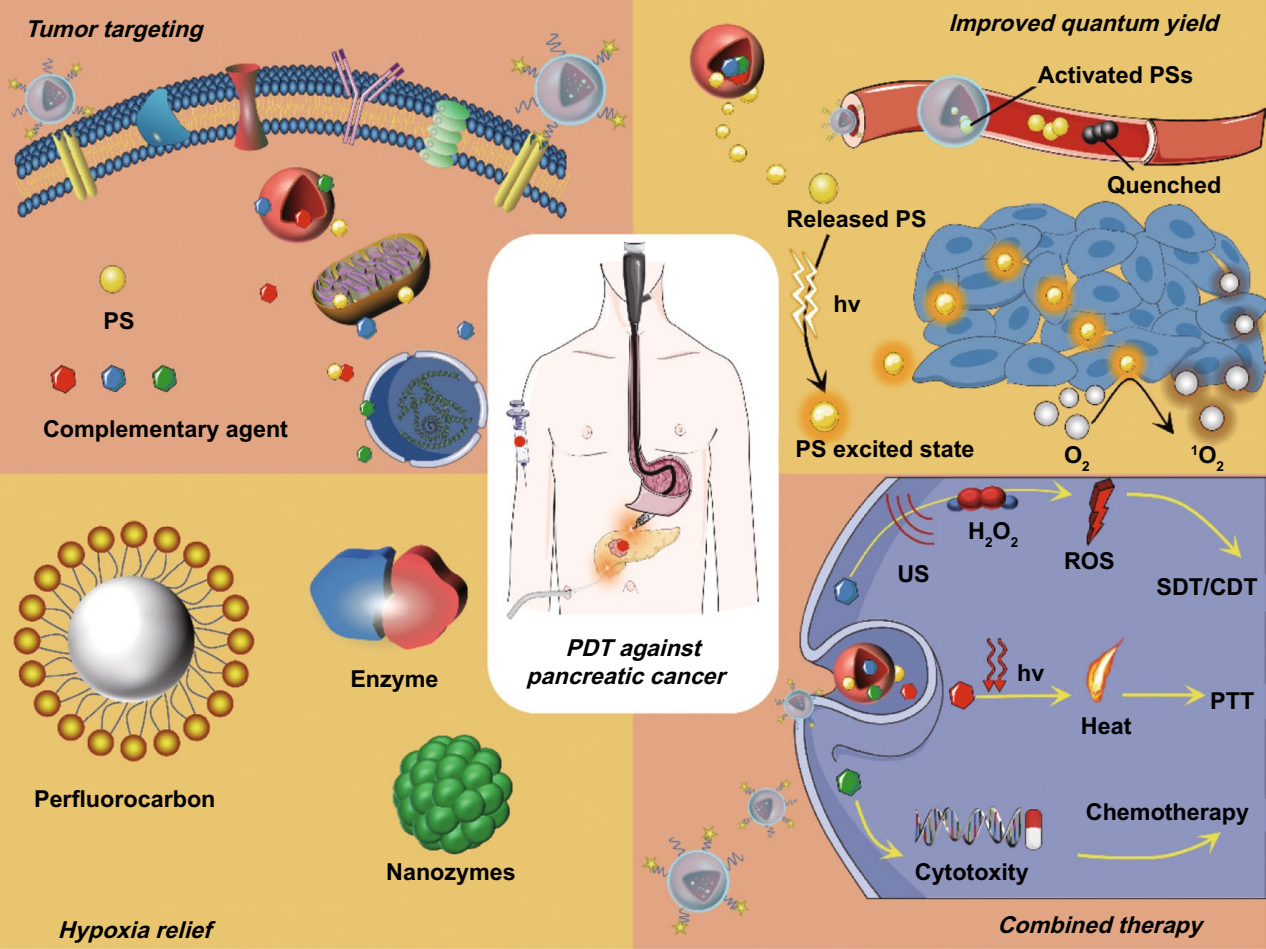
Schematic illustration of PS-loaded NPs for localized photodynamic destruction of PCa

However, PDT still suffers from certain limitations in these early phase studies. On the one hand, PDT against PCa is not completely free from severe adverse events such as gastrointestinal hemorrhage and duodenal obstruction, partly due to the poor selectivity of the existing PSs [7]. This nonselective distribution of PSs induces the risk of photodamage to the adjacent organs, and inefficient PSs accumulation in tumor sites. On the other hand, during follow-up after PDT, both liver metastases and tumor regrowth around edges of treated area occurred in some cases [12, 15]. The underlying reason may be related to the insufficient ^1^O_2_ yield within tumor. Conventional PSs such as porphyrins and other tetrapyrrole derivatives have poor water solubility and are prone to aggregate in physiological solutions through π–π stacking and hydrophobic interaction [7, 18]. This aggregation can severely impair ^1^O_2_ yield via aggregation caused quenching (ACQ) effect [19]. Moreover, single-modal PDT is not enough to cure PCa and combination with other therapy such as chemotherapy is critical to prolong the survival rate of Pca [20–22]. Thus, various kinds of PS-loaded nanoparticles were developed in the aim of overcoming these limitations (Fig. 1).

## Nanoparticles-Mediated PDT for Pancreatic Cancer

Encapsulating PSs in NPs can improve the solubility and stability of PSs, avoid self-quenching, and thereby increase ^1^O_2_ yield [23–25]. In addition, NPs can also be designed to deliver oxygen or generate oxygen in situ, thereby relieving tumor hypoxia, which is detrimental for efficient PDT [26–28]. The NPs can inherently target to tumors through the enhanced permeability and retention (EPR) effect, a unique phenomenon of solid tumors including PCa related to their anatomical and pathophysiological differences from normal tissues [29]. Moreover, the NPs can also be modified with specific ligands to achieve active tumor targeting [30].

### Improving Solubility of PSs and ^1^O_2_ Yield

**Fig. 2:**
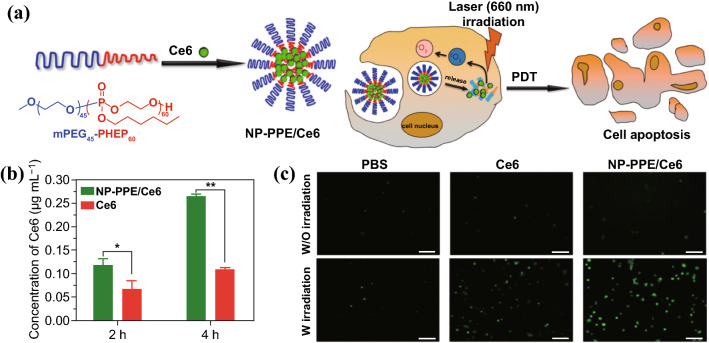
**a** Schematic illustration of Ce6 encapsulation by self-assembly of amphiphilic mPEG-b-PHEP and intracellular delivery of Ce6 with NP-PPE. **b** Quantitative Ce6 concentration in the BxPC-3 cancer cells. **c** CLSM image of cells incubated with DCF and then treated with free Ce6 and NP-PPE/Ce6 with NIR laser irradiation. Adapted with permission from Ref. [36]. Copyright © 2015, American Chemical Society

Most PSs such as porphyrins and other tetrapyrrole derivatives are hydrophobic and contain big heteroaromatic rings, which make PSs prone to aggregate by π–π stacking and hydrophobic interaction [7, 18]. By rational design of the PS encapsulation, PSs can be loaded with very high loading content while avoiding self-quenching [31–33]. Liang et al. fabricated a series of PS-loaded nanoparticles based on porphyrin-grafted lipid (PGL) [32, 34, 35]. The porphyrin molecules can be loaded in the PGL nanoparticles with a drug loading as high as 38.45% [32]. The orderly arranging mode of porphyrins and alkyl chains in the PGL molecules prevents PSs from aggregating and self-quenching, even at a very high density of PSs. Compared with the free porphyrin molecules, the generation of ^1^O_2_ by PGL nanoparticles in aqueous solution was elevated by several fold. Ding et al. developed a polyphosphoester-based nanocarrier (NP-PPE) to deliver Ce6 for PDT of PCa [36]. NP-PPE/Ce6 was prepared by self-assembly of amphiphilic diblock copolymer of methoxypolyethylene glycols (mPEG) and polyphosphoester, denoted as mPEG-b-PHEP, and Ce6 was loaded in the hydrophobic polyphosphoester core (Fig. 2). NP-PPE/Ce6 kept Ce6 in encapsulated state during circulation, but rapidly released Ce6 in the acid endosome or lysosome within cancer cells due to decreased hydrophobicity of polyphosphoester core. The ability of ^1^O_2_ generation of NP-PPE/Ce6 or free Ce6 was assessed by fluorescence intensity of the oxidized product dichlorofluorescein (DCF). As expected, NP-PPE dramatically improved accumulation of Ce6 and ^1^O_2_ generation in tumor relative to that of free Ce6 molecules, and NP-PPE/Ce6-mediated PDT treatment showed an enhanced antitumor efficacy on human BxPC-3 PCa-bearing mice.

### Oxygen Supplying

Oxygen is a key requirement for ^1^O_2_ generation in PDT and the efficacy of PDT can be compromised by hypoxic tumor microenvironment (TME). An important application of NPs is to establish an oxygen self-sufficient PDT nanoplatform for treating hypoxic PCa, which is characterized by a dense desmoplastic stroma as well as hypovascular and hypoperfused tumor vessels [37]. Oxygen can be loaded in NPs using oxygen-absorbing materials, such as liquid perfluorocarbon [38]. On the other hand, various in situ oxygen production nanoparticles, partially relying on the overproduced H_2_O_2_ in TME, are conceived as an alternative method to improve tumor oxygenation during PDT [28, 39].

**Fig. 3:**
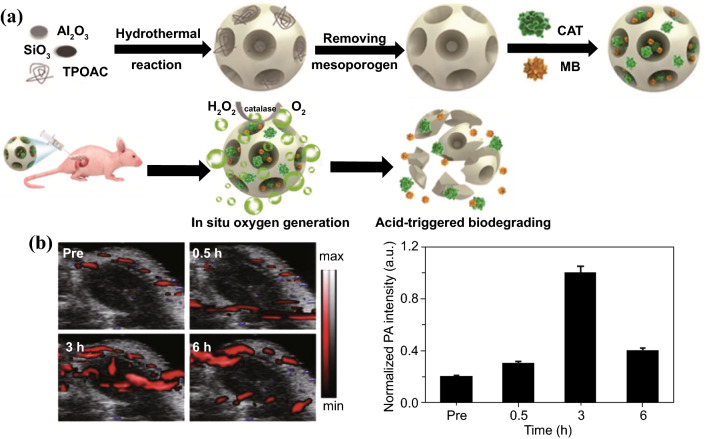
**a** Paradigm of the fabrication of mesoporous hierarchical zeolite nanocarriers loading catalase and MB characterized with oxygen generation and acid-triggered degradation. **b** *In vivo* 2D photoacoustic images and quantitative results of the blood oxyhemoglobin saturation in the tumor region at different time points after the intratumoral injection of the ZCM nanocapsule. Reproduced from Ref. [40] with permission. Copyright © The Royal Society of Chemistry 2018

A recent study by Hu et al. is a good example of NPs-based oxygen self-sufficient PDT platform, which is fabricated by loading catalase and methylene blue (MB) in mesoporous hierarchical zeolite nanocarriers (Fig. 3) [40]. Catalase loaded in the zeolite could efficiently relieve tumor hypoxia by continuously decomposing endogenic H_2_O_2_ and in situ producing a large amount of O_2_ inside tumor, thereby promoting efficacy of O_2_-dependent PDT. As shown by 2D photoacoustic imaging for oxygenated hemoglobin (*λ* = 850 nm), after the intratumoral injection of the zeolite-catalase-MB (ZCM) nanocapsule, the blood oxyhemoglobin level in tumor tissues increased over time and reached its maximum after 3-h post-injection. Moreover, the zeolite nanocarrier can be degraded under acid conditions, exhibiting high biocompatibility and biodegradability. In addition to natural catalase, nanozymes, synthetic nanomaterials with inherent enzyme-like characteristics, can also be designed with a range of enzymatic activity and have been explored as potential solutions to ameliorate tumor hypoxia during PDT. Kang et al. prepared a hollow Ru-Te nanorod (RuTeNR) with inherent oxidase, peroxidase, superoxide dismutase (SOD), and catalase-type activity, which also exhibited photothermal and photodynamic combinatorial effect under near-infrared (NIR) laser irradiation [41]. This RuTeNR was synthesized through solvothermal galvanic replacement of the sacrificial Te nanorod template with Ru(III) as the replacement metal cation. By the peroxidase-like activity, RuTeNRs decomposed H_2_O_2_ in TME into hydroxy radicals (HO^·^) exerting cytotoxic effect. Meanwhile, RuTeNRs converted H_2_O_2_ into O_2_ by their catalase-like activity to promote ^1^O_2_ generation of PDT. Under photoactivation, RuTeNRs-mediated PDT effect synchronized with their photothermal effect through segregated photonic pathways in suppressing MIA PaCa-2 pancreatic tumors.

**Fig. 4:**
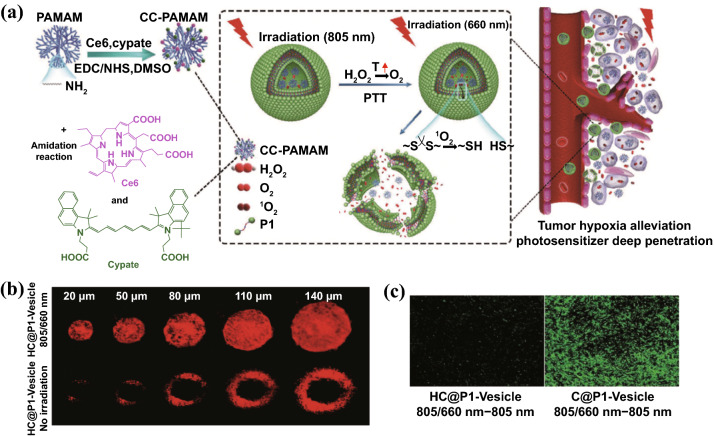
**a** Schematic illustration of light-triggered clustered polymeric vesicles with self-supplied oxygen and tissue penetrability for potent PDT against hypoxic PCa tumor. **b** Penetration profiles of CC-PAMAM after HC@P1-Vesicle treatment with or without 805/660 irradiation in multicellular tumor spheroids. The imaging was conducted at 2-h postirradiation using CLSM. **c** Hypoxia of BxPC-3 tumor treated with C@P1-Vesicle or HC@P1-Vesicle using a hypoxia-specific probe pimonidazole (hypoxyprobe-1 plus kit). HC@**P1**-Vesicle: H_2_O_2_ and CC-PAMAM-loaded ROS-responsive **P1** polymeric vesicles. C@**P1**-Vesicle: CC-PAMAM-loaded **P1** polymeric vesicles without H_2_O_2_. Reproduced from Ref. [42] with permission. Copyright © 2017 WILEY-VCH Verlag GmbH & Co. KGaA, Weinheim

In another significative study by Li et al. [42], the tumor hypoxia alleviation strategy relied on photothermal effect to decompose H_2_O_2_ into O_2_. In detail, Ce6/cypate-conjugated poly(amidoamine) dendrimers (CC-PAMAM) and H_2_O_2_ were co-loaded within ROS-responsive polymeric vesicles (Fig. 4). Upon 805 nm laser irradiation, the heat generated by photothermal effect of cypate decomposes H_2_O_2_ into O_2_, alleviating hypoxic TME in tumor site, as evidenced by weak fluorescence intensity of hypoxia-specific probe pimonidazole and less than 10% hypoxic areas. Followed by 660 nm irradiation, Ce6 produced abundant ^1^O_2_ with the assistance of self-supplied oxygen. Then, the generated ^1^O_2_ could disrupt the ROS-responsive polymeric vesicles, which subsequent triggered CC-PAMAM diffusing out from vesicular chamber. The released CC-PAMAM was able to penetrate tumors forming a uniform distribution for photodynamic ablation of tumor cells. The involved light treatment procedure comprised five cycles of consecutive irradiations with 805 nm light for 3 min and 660 nm light for 10 min at 24 h post-CC-PAMAM/H_2_O_2_ vesicles injection, leading to complete elapse of the BxPC-3 PCa without any regrowth.

The dense collagen network in PCa stroma strongly hinders the intratumoral penetration of oxygen and drugs. Losartan and other angiotensin receptor blockers (ARBs) have been proven to inhibit tumor collagen production via downregulation of TGF-β1 [43]. In the AK4.4 pancreatic tumors, losartan treatment was shown to increase drug accumulation by 74% and oxygen delivery by over onefold. Thus, depletion of collagen with ARBs is a promising strategy to enhance delivery efficiency of PSs and alleviate hypoxia in pancreatic cancer, thereby improving the PDT efficacy. In a study conducted by Li et al. [44], pretreatment with losartan increased the accumulation of PS-loaded nanoparticles by twofold and the tumor growth rates were obviously delayed.

### Tumor Targeting

NPs can be targeted to tumor tissues via EPR effect owing to their leaky vasculature and poor lymphatic drainage [45]. In addition, NPs can also be decorated with tumor targeting ligands, such as monoclonal antibodies, antibody fragments, and small molecules, for further enhanced tumor targeting [46–48]. Conjugation to specific ligands or surface receptors in PCa that allows targeted delivery of photosensitizer to PCa cells will remarkably improve the PDT efficacy of PCa treatment. Some potential targets have been suggested for the specific delivery in PCa, such as EGFR, transferrin, epithelial cell adhesion molecule (EpCAM), CD44, CD133, urokinase plasminogen activator receptor (uPAR), ERBB2, and CA125 [49–52]. Er et al. used cetuximab (Cet)-modified mesoporous silica nanoparticles (MSNPs) for the tumor targeting delivery of PSs [53]. Cet is a monoclonal antibody of EGFR (epidermal growth factor receptor), which is overexpressed on several PCa types. The Cet decoration facilitates cell recognition and internalization of MSNPs during treatment, which in turn accelerates the release of PSs within cells. A similar Cet-mediated PDT was reported by Obaid et al. [54]. Cet was conjugated on the liposomal formulation of PSs-anchored phospholipid via site-specific Protein Z tuning approach, and the obtained photoimmuno-nanoconjugates (PINs) was denoted as Cet-PINs. By optimizing PS lipid anchoring, surface electrostatics, Cet surface orientations, and Cet densities, ~ 16-fold enhancement in binding specificity and targeted photodestruction was achieved. The performance of the Cet-PINs was studied on a stroma-rich heterotypic xenograft model consisting of MIA PaCa-2 cells and pancreatic cancer-associated fibroblasts. Due to the actively homing effect of Cet, the BPD-delivering Cet-PINs achieved rapidly penetration up to 470 μm away from vessels within 1 h in heterotypic PCa organoids. Additionally, Cet-PINs-mediated PDT exhibited excellent tumor-targeted photodestruction efficiencies, including a 1.5-fold reduction in tumor collagen density and a statistically significant ~ 3-fold increase in fractional necrotic area at 72-h post-treatment. Remarkably, the used BPD equivalent dose of this Cet-PIN formulation is ~ 10-fold lower than that of clinically approved liposomal formulation verteporfin, underscoring the value of molecular targeted PS delivery.

## Combining PDT with Other Therapies

Although PDT is able to kill cancer cells under the optimized condition, tumor relapse is unavoidable in practical scenario with PDT alone. This can be explained by incomplete elimination of cancer cells partly due to the heterogeneous distribution of PSs and light in tumor tissues [42]. In addition, the exacerbated hypoxia induced by PDT also stimulates several signaling pathways leading to cancer cell survival and escape [55]. Therefore, there is a critical need to combine other therapy with PDT for maximized therapeutic efficacy. NPs hold excellent capability to integrate various therapeutic agents together with PSs for PDT-based combinatory treatment.

### Combining PDT with Photothermal Therapy

Photothermal therapy (PTT) makes use of photothermal agents that can convert the absorbed light energy into heat to increase the temperature of surrounding environment and trigger the death of cancer cells [56–58]. The combined therapy with PDT and PTT is attractive in treating cancer, because the cytotoxic ^1^O_2_ and hyperthermia are produced by PDT/PTT sensitizers in similar light triggering conditions [59, 60]. Besides, many light-responsive nanomaterials can absorb photons from NIR laser irradiation and then generate both heat and ^1^O_2_ relying on different non-fluorescent excited state relaxation pathways that can function in parallel [61, 62]. Examples of such dual functional materials include inorganic species (e.g., AuNPs, black phosphorus, graphene oxide, and Ti_3_C_2_ nanosheets) and organic species (e.g., diketopyrrolopyrroles, heptamethine dyes (indocyanine green, IR780, IR825, IR808, IR2), and metallonaphthalocyanines) [59, 61, 63, 64]. Moreover, the photothermal conversion is oxygen-independent, which makes PTT an excellent compensate for oxygen-dependent PDT, especially in hypoxic tumors [65]. However, PTT alone is unlikely to eliminate all tumor cells because the resulting heat can be rapidly dissipated by circulating blood [66, 67]. Therefore, combined PTT/PDT treatment can enhance the outcome of cancer therapy by compensating for the drawbacks of monotherapies [68].

*Yin* and coworkers developed the biodegradable PEGylated oxygen-deficient molybdenum oxide nanoparticles (PEG-MoOx NPs), which could effectively convert light into heat and generate ^1^O_2_ simultaneously with a single 1064 nm NIR irradiation [69]. The PEG-MoOx NPs were synthesized through a one-pot hydrothermal process using PEG-4000 and (NH_4_)_6_Mo_7_O_24_·4H_2_O hydrolyzed in water–ethanol solution (pH = 1.2). The mechanism of ^1^O_2_ production was speculated to be increased thermionic electron emission of PEG-MoOx NPs caused by extreme heat development upon 1064 nm NIR activation. The synergistic PDT/PTT effect of PEG-MoOx NPs with 1064 nm irradiation caused a far better tumor therapy effect in PANC-1 bearing mice at a lower light dose (0.6 W cm^−2^), compared with sole PTT effect of PEG-MoOx NPs induced by 808 nm laser (0.75 W cm^−2^).

Li et al. applied a distinct strategy for combinatorial PDT/PTT treatment against PCa, wherein gold nanoparticles (AuNPs) were employed to not only deliver PSs but also act as photothermal agents [70]. The photosensitizer prodrug 5-aminolevulinic acid (5-ALA) was linked with peptide CRQAGFSL, which can be cleaved by tumor-specific intracellular CathepsinE (CTSE), to realize targeting release of PSs within tumor. The CRQAGFSL-5-ALA conjugated AuNPs were cross-linked with 1,9-nonanedithiol forming spherical gold nanoclusters. In addition, an active targeting U11 peptide was also conjugated on the surface of nanocluster. This multifunctional nanocluster-based platform represents a promising PDT/PTT agent for highly synergistic therapeutic effect against PCa with reduced side effects in normal pancreas tissues.

### Combining PDT with Other ROS-Generating Therapy

For ROS-dependent anticancer treatment, a relatively high level of ROS is required to induce irreversible oxidative damage, because cancer cells can protect themselves from oxidative stress through the overexpressed intracellular reducing thiol species [71]. However, the ROS generation efficiency of PDT is largely limited to the low tissue oxygen in pancreatic cancers. In addition, PSs widely used in clinic generally absorb light in the visible red range [72]. Even though NIR light can penetrate deeper than visible light, it can only reach around 3 cm deep in tissue [59]. This limits clinical applications of PDT, especially in large tumors. The combination of PDT with other ROS-generating therapy is a promising approach for enhanced ROS-mediated therapeutic outcome.

**Fig. 5:**
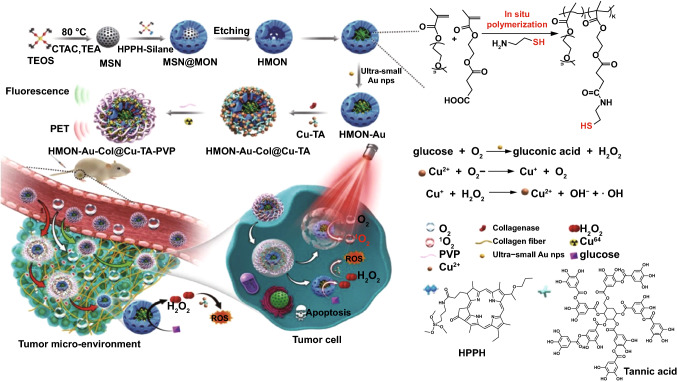
Schematic showing the fabrication process of HMONs-Au@Cu-TA and its application for synergistic PDT/CDT therapy. Reprinted from Ref. [44] with permission. Copyright © 2019 WILEY-VCH Verlag GmbH & Co. KGaA, Weinheim

Recently, the combination of PDT and chemodynamic therapy (CDT) has been continuously explored to amplify the tumor oxidative stress [73, 74]. The therapeutic mechanism of CDT lies in the catalysis of metal ions-based nanoparticles to convert endogenous H_2_O_2_ into cytotoxic hydroxyl radicals (^·^OH) via Fenton-like reaction [39]. Although CDT is an unremitting chemical process overcoming the limitations of light attenuation and hypoxic TME [75], the efficacy of CDT is always restricted by the unsatisfactory catalytic efficiency of currently developed metal-based nanocatalysts [39]. The extent of acidity in TME also restricts the activity of these metallic catalysts, which obviously decreases CDT performance [76]. Additionally, the concentration of H_2_O_2_ in tumor tissue (< 100 μM) is still insufficient to generate enough ^·^OH for effective CDT [77]. For all of these, CDT alone still cannot achieve an envisioned therapeutic outcome in intractable PCa. However, the encouraging results have been obtained with dual-modal PDT/CDT with better therapeutic effect than CDT or PDT alone [78]. A recent study by Li et al. [44] is a good example of such combined PDT/CDT in treating PCa, where they employed hollow mesoporous organosilica nanoparticle (HMON) to co-deliver PDT/CDT agents so as to produce ^1^O_2_ and ^·^OH in response to laser irradiation and tumor H_2_O_2_ for enhanced antitumor effects. The photosensitizer HPPH was incorporated in the framework of HMON through cohydrolysis of HPPH-silane and silane precursors, and the hollow cavity of HMON was exploited as a nanoreactor for in situ polymerization to immobilize ultrasmall Au NPs (< 3.6 nm) via the chelation effect (Fig. 5). These ultrasmall Au NPs behaving like glucose oxidase could catalyze glucose into H_2_O_2_ to provide self-supplied H_2_O_2_ for CDT. CDT agents Cu^2+^-tannic acid (Cu-TA) complexes were deposited on the surface of HMON-Au, which can catalyze the self-supplied H_2_O_2_ into ^·^OH in acidic TME. Collagenase (Col) was further loaded into the HMONs-Au@Cu-TA to degrade the dense stroma within PCa thereby enhancing the deep penetration of HMONs and promoting O_2_ infiltration to alleviate hypoxia. The combined PDT/CDT produced large amount of intracellular ROS in BxPC-3 cells and eliminated the tumors on BxPC-3 bearing mice, superior to either monotherapy.

Sonodynamic therapy (SDT) is another ROS-dependent treatment, referring to the use of low intensity ultrasound to activate sonosensitizers, which converts tissue oxygen to cytotoxic ^1^O_2_ [71, 79–81]. To date, almost all the sensitizers used in SDT based studies were originally used as photosensitizers [82]. Due to the higher tissues penetration of ultrasound, combining SDT with traditional PDT can make up the tissue penetration restriction of light. Besides, sono-photodynamic therapy (SPDT) can decrease the necessary dosage of sensitizer and light energy of PDT, which in turn further reduces its off-target photocytotoxicity [83]. Indeed, SPDT treatment using sono/photosensitizers which can be activated by light plus US irradiation has been tested with enhanced ROS generation and better therapeutic outcomes than SDT or PDT alone in various tumor types [84–87]. Wang et al. [86] investigated Chlorin e6 (Ce6)-mediated SPDT (Ce6-SPDT) on 4T1 cells and animal models. Much more ^1^O_2_ generation was observed in cells treated by SPDT compared with PDT or SDT alone (MFI = 156 in SPDT vs MFI = 51 in PDT/MFI = 10 in SDT, mean fluorescence intensity). In addition, Ce6-SPDT markedly inhibited tumor growth (volume and weight) and lung metastasis in 4T1 tumor-bearing mice. The synergistic effects of PDT/SDT observed in other types of cancer should be applicable to pancreatic cancer. A recent study by Chen et al. showed a rational design of HMONs-based oxygen-loaded nanoplatform for SDT treatment on PCa models [88]. The mesoporous structure of HMONs was served as IR780 carrier. Meanwhile, HMONs were chelated with fluorocarbon (FC)-chains, which served as oxygen binding sites for exogenous oxygen delivery. IR780 has been reported for producing ^1^O_2_ under irradiation with 808 nm laser [89]. Therefore, theoretically, this IR780@O_2_-FHMON is promising for exerting IR780-mediated PDT/SDT synergistic effect, which can be further enhanced by self-supplying oxygen.

### Multimodal Synergistic Therapy Based on PDT

Given the aggressive nature from an early stage and the multiple escape mechanisms of pancreas cancer, any monotherapy is unlikely to cure cancer completely [90]. Thus, tumor relapse and metastasis occurred in several cases after sole PDT treatment [12, 15]. Thus, the appropriately conceived combination strategies based on PDT are essential for this extremely difficult-to-treat cancer, such that therapeutics targeting distinct tumor compartments or signaling pathways could induce synergistic effects to overcome stubborn PCa resistance.

The combination of chemotherapy and PDT has shown promising synergistic effect in various cancer types including PCa both in the preclinical and clinical studies [91–93]. Zhang et al. conjugated Fe^3+^ with photosensitizer 5,10,15,20-tetra(p-benzoato)porphyrin (TBP) to create a porous coordination network (PCN) [94]. This Fe(III)-TBP PCN was able to load paclitaxel (PTX) and release it in response to both laser irradiation and pH changes within tumors. Besides, catalase-like Fe^3+^ could catalyze endogenous H_2_O_2_ decomposed into O_2_ to support PDT activity. Thus, a single nanoplatform-mediated synergistic effect of chemo/PDT was achieved in PANC-1 tumors, where phase-blocked cells were accelerated in transforming into apoptotic state upon PDT introduction.

**Fig. 6:**
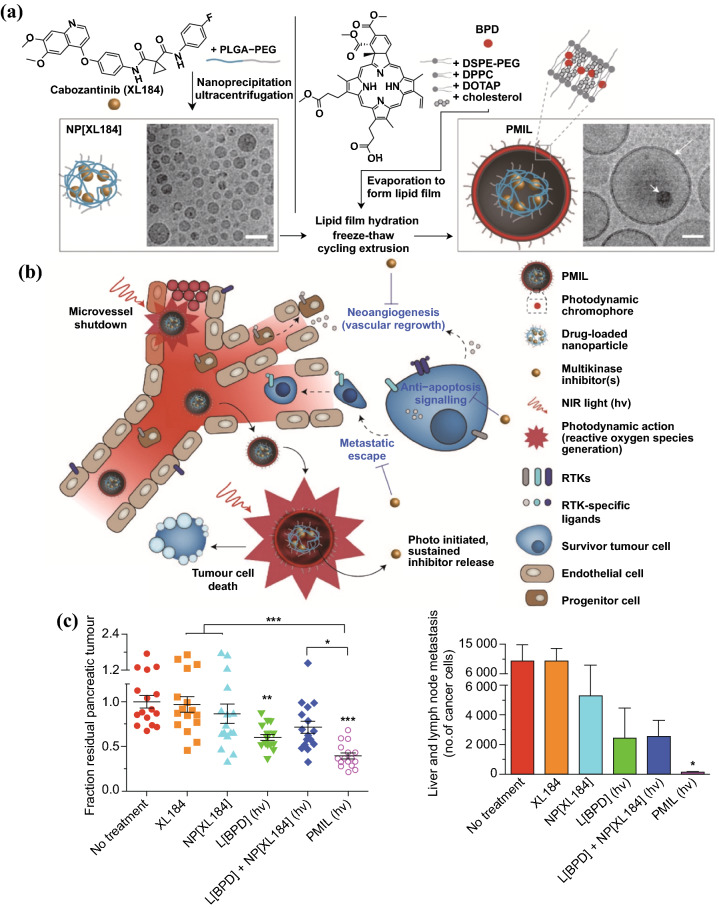
Schematic illustration of a fabrication of **a** photoactivatable multi-inhibitor nanoliposome (PMIL) and **b** its applications for combination therapy with PDT-induced tumor cell death and microvessel damage and inhibition of treatment escape pathways. **c** A single PMIL treatment in orthotopic PDAC mouse achieves enhanced reductions in primary (Left) and metastatic tumors (Right). Reproduced from Ref. [55] with permission. Copyright © 2016 Macmillan Publishers Limited

In addition to directly killing tumor cells, PDT can also induce microvascular shutdown, which contribute to exacerbated hypoxia in TME and nutrients deprivation. Although the vascular damage can induce tumor death, the increased tumor hypoxia also stimulates several signaling pathways leading to angiogenesis and tumor metastasis [5, 95]. To solve this issue, Spring et al. introduced a photoactivatable multi-inhibitor nanoliposome (PMIL) for combining PDT with cabozantinib (XL184), a multikinase inhibitor, which targets vascular endothelial growth factor (VEGF) signaling and MET—the receptor tyrosine kinase for hepatocyte growth factor—signaling (Fig. 6) [55]. VEGF signaling promotes tumor angiogenesis and vascular regrowth, while MET signaling supports cancer cell survival and promotes cancer cell metastatic escape from hypoxic tumor induced by cytotoxic and vascular damage therapy [96]. PMIL is fabricated by encapsulating XL184-loaded poly(lactic acid-co-glycolic) acid (PLGA) NPs in BPD-loaded cationic liposome. Upon irradiation with 690 nm laser, the light-activated ROS generation by BPD induced not only tumor cell death and microvessel damage, but also liposomal disruption to release XL184-loaded PLGA NPs. The PLGA NPs were designed for sustained release of XL184 over a period of several days. XL184 inhibits both the VEGF and MET signaling pathways to suppress tumor escape. The prominent therapeutic efficacy of PMIL was validated on two PCa xenograft model with significantly enhanced inhibition in both primary and metastatic tumor.

## Conclusion and Outlook

The efficacy of PDT on alleviating tumor burden in unresectable localized PCa patients have been validated in several clinical trials, with relatively lower complications [12, 15–17]. Under the guidance of ultrasound imaging and/or CT, optical fibers can be inserted into the pancreas tumors in a percutaneous or transduodenal approach to realize minimal-invasive photodynamic tumor ablation. Despite the promising results obtained in the preliminary clinical trials, the efficacy of PDT is largely compromised by lack of suitable PSs in terms of tumor targeting, ^1^O_2_ yield, and adaptability to hypoxic TME. In this regard, NPs-based PSs have shown the promising results to solve these issues in various preclinical researches. Rationally designed nanocarriers can load PSs with very high loading content while avoiding potential aggregation and improving ^1^O_2_ generation [31–33]. To further improve the ^1^O_2_ generation in hypoxic tumor, PSs-loaded NPs can also be designed for oxygen delivery or in situ oxygen generation [38]. In addition, the NPs inherently can be targeted to tumors via EPR effect or modified with specific ligands to achieve active tumor targeting [41]. Moreover, since PDT alone is not enough to cure PCa completely, various therapeutic agents can also be co-loaded with PSs in NPs to synchronize with PDT for maximized therapeutic efficacy [5, 55, 95].

Although NPs-based PDT have achieved far better therapeutic efficacy than conventional PSs for PCa therapy in the preclinical studies, the translation of NPs-based PDT from bench to bedside is not straightforward. There are still several issues to address for the clinical translation of NPs-based PDT. On the one hand, various aspects in the NPs design need to be optimized in respect to PSs loading, surface charge, size, targeting modification, etc. [54], as these factors determine the extent of interactions between NPs and biomolecules, and are crucial in pharmacology and clearance rate of NPs. On the other hand, although integrating various therapeutic components in one NP benefits cancer therapy in preclinical studies, their usually sophisticated manufacturing process may complicate the potential pharmaceutical development in terms of quality control and reproducibility. In addition, most of the preclinical studies are conducted in tumor xenograft model of mice and there is still a lack of researches on large animals or other clinical-relevant models. The potential safety issues and consistency of therapeutic efficacy are the major concerns. For the purpose of successful clinical translation of synthesized NPs, the detailed biodistribution investigation and excretion assay are highly desired to evidence their good biocompatibility and biodegradability. Repeated permutation experiments for certain NPs should be carried out to establish optimum treatment parameters such as light dose, interval time between PS administration and irradiation, PS dose and administration method, so as to guide future clinical translation. Collectively, the present studies have demonstrated the potential of NPs-based PDT for PCa therapy and further studies are warranted to optimize NPs design and investigate the long-term safety and efficacy. The existing clinical studies and continuing phase II/III studies of PDT in PCa form a good basis for developing NPs-based PDT against PCa.
